# Healthcare-Associated Infections Are Associated with Insufficient Dietary Intake: An Observational Cross-Sectional Study

**DOI:** 10.1371/journal.pone.0123695

**Published:** 2015-04-29

**Authors:** Ronan Thibault, Anne-Marie Makhlouf, Michel P. Kossovsky, Jimison Iavindrasana, Marinette Chikhi, Rodolphe Meyer, Didier Pittet, Walter Zingg, Claude Pichard

**Affiliations:** 1 Nutrition Unit, Geneva University Hospital, Geneva, Switzerland; 2 Rehabilitation and Geriatrics, Geneva University Hospital, Geneva, Switzerland; 3 Business Intelligence Division, Geneva University Hospital, Geneva, Switzerland; 4 Department of Informatics, Geneva University Hospital, Geneva, Switzerland; 5 Infection Control Programme, Geneva University Hospital, Geneva, Switzerland; University of Leicester, UNITED KINGDOM

## Abstract

**Background:**

Indicators to predict healthcare-associated infections (HCAI) are scarce. Malnutrition is known to be associated with adverse outcomes in healthcare but its identification is time-consuming and rarely done in daily practice. This cross-sectional study assessed the association between dietary intake, nutritional risk, and the prevalence of HCAI, in a general hospital population.

**Methods and findings:**

Dietary intake was assessed by dedicated dieticians on one day for all hospitalized patients receiving three meals per day. Nutritional risk was assessed using Nutritional Risk Screening (NRS)-2002, and defined as a NRS score ≥ 3. Energy needs were calculated using 110% of Harris-Benedict formula. HCAIs were diagnosed based on the Center for Disease Control criteria and their association with nutritional risk and measured energy intake was done using a multivariate logistic regression analysis. From 1689 hospitalised patients, 1024 and 1091 were eligible for the measurement of energy intake and nutritional risk, respectively. The prevalence of HCAI was 6.8%, and 30.1% of patients were at nutritional risk. Patients with HCAI were more likely identified with decreased energy intake (i.e. ≤ 70% of predicted energy needs) (30.3% vs. 14.5%, P = 0.002). The proportion of patients at nutritional risk was not significantly different between patients with and without HCAI (35.6% *vs*.29.7%, P = 0.28), respectively. Measured energy intake ≤ 70% of predicted energy needs (odds ratio: 2.26; 95% CI: 1.24 to 4.11, P = 0.008) and moderate severity of the disease (odds ratio: 3.38; 95% CI: 1.49 to 7.68, P = 0.004) were associated with HCAI in the multivariate analysis.

**Conclusion:**

Measured energy intake ≤ 70% of predicted energy needs is associated with HCAI in hospitalised patients. This suggests that insufficient dietary intake could be a risk factor of HCAI, without excluding reverse causality. Randomized trials are needed to assess whether improving energy intake in patients identified with decreased dietary intake could be a novel strategy for HCAI prevention.

## Introduction

Malnutrition is associated with increased mortality and morbidity, extends hospital length of stay, reduces quality of life, and increases the healthcare costs [1–6]. Systematic screening at hospital admission by using the Nutritional Risk Screening-2002 score is recommended to detect patients at nutritional risk [7]. The data about the relation between dietary intake and clinical outcome are scarce. The European multicentre observational survey, NutritionDay, performed in 16,290 patients hospitalised from 25 countries, has shown that intake below 25% of provided food was associated with increased in-hospital mortality [8]. Agarwal et al. demonstrated that malnutrition and insufficient dietary intake were associated with longer hospital length of stay, more readmissions, and a higher mortality rate [9]. Recently, Tangvik et al showed in 3279 Norwegian hospitalised patients that reduced dietary intake was associated with a doubling of one year mortality [10]. Patients with poor nutritional status had more healthcare-associated infections (HCAI) than those with normal nutritional status [5,11]. In intensive care patients, energy deficit during the first week of stay is correlated with an increased proportion of infections [12]. Timely and targeted nutritional interventions showed a reduced incidence of HCAI in perioperative and intensive care patients [13–15]. The burden of HCAI is high, as well as its economic impact. In US hospitals, it has been estimated that the prevention of HCAI could reduce the HCAI hospital costs by several billion US $, e.g. economy of two to three billion US $ for the prevention of ventilator-associated pneumonia [16]. The prevention of HCAI has become a hospital standard in terms of quality management and safety of patients.

This study aims at assessing the association between dietary intake, nutritional risk, and the prevalence of HCAI, in an unselected hospital population. Our hypothesis is that patients with insufficient dietary intake are at increased risk for HCAI. If proven, detecting hospitalised patients with insufficient energy intake to initiate an early nutritional intervention would help to reduce HCAI.

## Materials and Methods

**Fig 1:**
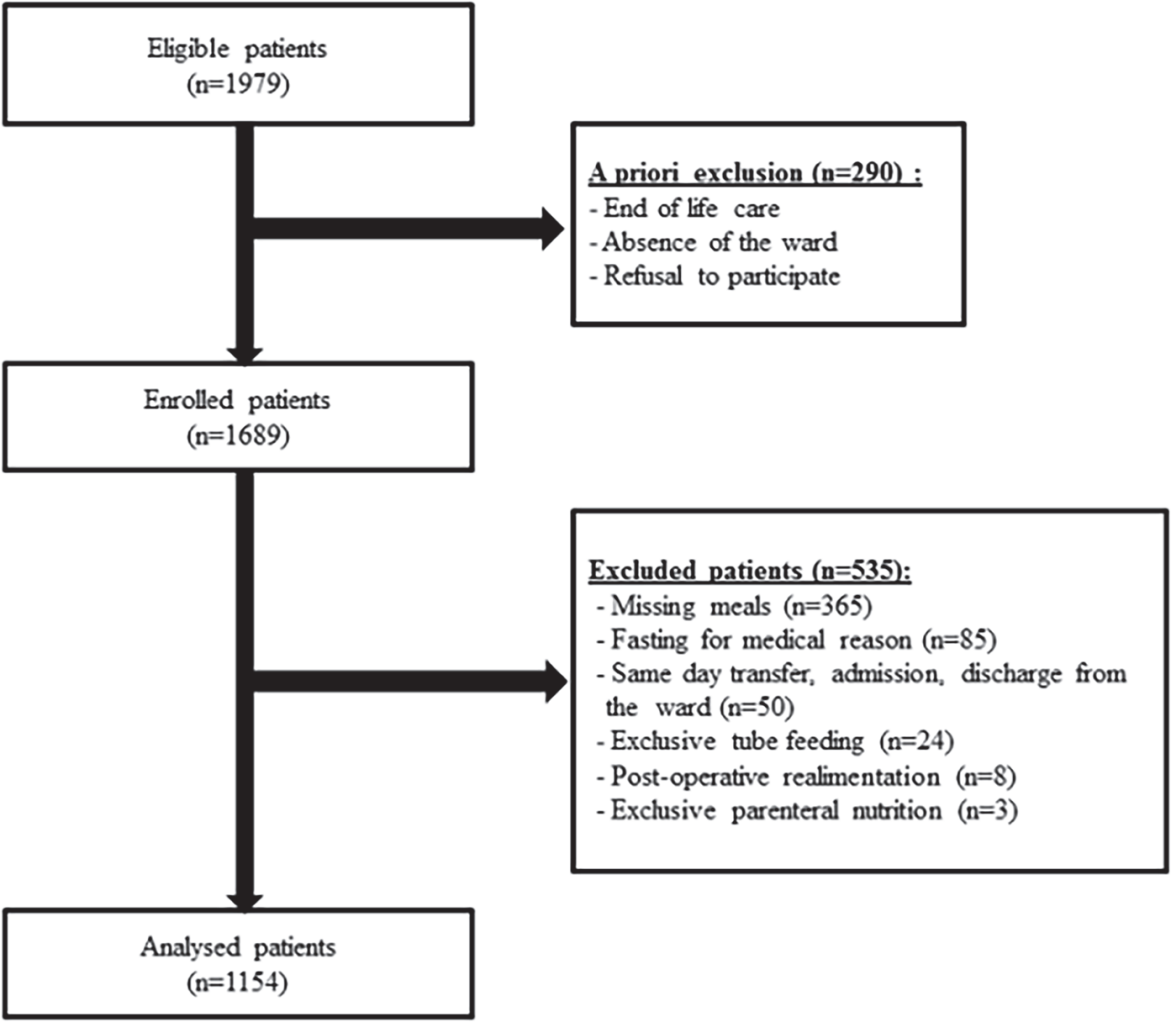
Study flow chart.

The Geneva University Hospital is the largest University-affiliated primary and tertiary referral centre in Switzerland. All types of care are represented: medicine, surgery, rehabilitation, psychiatry, and long term facility. The study was performed on one single day on adult patients of all departments, except the intensive care unit, between September 27th and November 20th 2012. All hospitalised patients were eligible. Exclusion criteria were end-of-life care, exclusive tube feeding or parenteral nutrition, and missed meals due to fasting for medical reasons, death, or transfer, admission and discharge from the ward (Fig 1). As a routine procedure at the Geneva University Hospital, patients select their menus which are served on individual trays three times a day. The assessment of dietary intake was standardized and performed by a team of 109 well-trained dieticians. All dieticians, including students of the Geneva Dietetic School were trained before the assessment with the same teachers (AMM, MC, CP). For at-bed measurement of dietary intake, all students were supervised by qualified dieticians who received the same information regarding the methodology of dietary intake. Dietary intake was calculated by analysing the differences between consumed and provided meals, snacks, oral nutritional supplements, supplemental tube feeding and parenteral nutrition. The energy from dietary intake was calculated for each meal using the dietary service software Winrest (FSI, Noisy-le-Grand, France) for which a training was done. The predicted energy needs were calculated as previously shown [17,18], according to the current ESPEN recommendations [19,20]. Energy needs were calculated with the Harris-Benedict formula increased by 10% to cover increased needs due to hospitalization and disease (e.g. stress, fever, digestive or renal losses). Predicted protein needs were calculated as 1.2 or 1.0 g/kg/day for patients < 65 or ≥ 65 years respectively. [19,20]

The nutritional risk was assessed using the validated Nutritional Risk Screening-2002 score [7]. This score is stratified into three parts addressing nutritional status, disease severity, and age. A first scoring between zero and three is attributed to impaired nutritional status (score 0 = absent, score 1 = mild, score 2 = moderate, score 3 = severe) based on two different items: percentage of weight loss and/or value of body mass index. A second scoring between zero and three points is dedicated to the disease severity from absent (zero) to severe (three). A last scoring is attributed to age: one if >70 years, zero if ≤ 70 years. Patients were considered at nutritional risk if the final Nutritional Risk Screening-2002 score was ≥ 3. In addition, patients with weight loss ≥ 5% within the last three months were identified.

Data on fever (≥38°C) and antibiotics were obtained from the patient charts on the study day. These parameters were served as case finding determinants for detailed HCAI screening. Former data analysis (2010) of the Geneva University Hospital has shown that more than 93% of patients with HCAI had received antibiotics or had fever before and on the day of prevalence. HCAI were identified by using the methodology of the point prevalence [21]. HCAI definitions were based on the Centres for Disease Prevention and Control [22] and surveillance was done by an infection control physician (WZ), who was blinded for the measurement of dietary intake. Infection types were classified into several categories: urinary tract infection, lower respiratory tract infection including pneumonia, surgical site infection, bloodstream infection, gastrointestinal system infection and other. Other infections included cardiovascular system infection, eye, ear, nose, throat or mouth infection, reproductive tract infection, and skin and soft tissue infection. The admission date, to calculate time to-survey, and the main diagnoses were retrieved from the electronic database of the hospital. Diagnoses were classified into six categories: ear nose throat-nervous system, internal medicine (including endocrine, gastrointestinal, haematological and skin diseases), orthopaedic, psychiatry, thorax (including heart, lung and vascular diseases), and obstetrical and urogenital diseases.

The Ethical Committee of the Geneva University Hospital approved this study and waived the need for a written consent as the study was part of a continuous general audit in our institution for the improvement of the quality of care programme. All patients were informed about the study by a written document delivered on their meal tray, and could refuse to participate in it.

### Statistical analysis

All statistical analyses were conducted by Stata software, release 12.0 (Stata Corporation, College Station, Texas, USA). Shapiro-Wilk test was used to evaluate the normality of variables. Categorical variables were described by numbers and percentages, and continuous variables by mean (±standard deviation) or median [extremes]. The locally weighted scatterplot smoothing (lowess) graphical procedure was used to determine a threshold of measured energy intake below which the risk of HCAI increased [23]. This method fits simple models to localized subsets of the data to build up a function and was selected because no global function to fit a model to the data needed to be specified. Even though this method does not produce a regression function that can easily be expressed by a mathematical formula, its flexibility makes it ideal for modeling complex processes for which no theoretical models exist. In order to determine whether measured energy and protein intakes ≤ 70% of predicted needs were associated with HCAI, multivariate logistic modelling was performed adjusting for age, gender, Nutritional Risk Screening-2002 score items (impaired nutritional status, and severity of the disease), time from admission to the day of prevalence, speciality ward (medicine, surgery, rehabilitation—psychiatry—long term facility) and the presence or absence of cancer. Statistical significance was set for a two-sided *P*-value < 0.05.

To estimate the money saving in case of the hypothesis that increasing energy intake from ≤70% to >70% would have reduced the number of HCAI, we propose a financial simulation. The results obtained from our study population were transposed to the whole Geneva University Hospital adult patients hospitalized in acute care departments in 2012, using the 2012 Swiss-DRG data retrieved from the Department of Informatics of the Geneva University Hospital. The proportion of patients with HCAI was calculated in the subgroups of patients having measured energy intake ≤ 70% and >70% of predicted energy needs. The expected reduced rate of HCAI was calculated according to the formula: [(B-A)/A]. The expected numbers of HCAI diagnosed and saved were deduced. The estimation of money saving was calculated according to CDC data [24] based on the low and high estimates of average HCAI attributable costs, 14,000 and 15,300 US $/patient, respectively.

### Ethical approval

The Ethical Committee of the Geneva University Hospital approved this study and waived the need for a written consent as the study was part of a continuous general audit in our institution for the improvement of the quality of care programme. All patients were informed about the study by a written document delivered on their meal tray, and could refuse to participate in it.

## Results

Out of 1689 enrolled patients, 1154 were analysed for HCAI (Fig 1). Exclusion occurred mostly because one or more missing meals (365/535 (68.2%)) and fasting for medical reasons (85/535 (15.9%)). Because of missing data, 1024 patients were analysed for energy intake, and 1091 for nutritional risk. Patients with HCAI were more frequently admitted for a surgical, internal medicine, and obstetrical-urogenital diagnoses (Table 1). HCAI prevalence was 6.8% (79/1154). The most common HCAI was urinary tract infection (24/79) followed by pneumonia (18/79) (Table 1). Compared to non infected patients, patients with HCAI had prolonged hospital length of stay and higher all-cause hospital mortality (Table 1). There was a trend towards more HCAI among patients with weight loss ≥ 5%.

**Table 1:** Patient characteristics and clinical diagnoses, according to the presence or absence of healthcare-associated infections (HCAI).

Variables	Presence of HCAI	Absence of HCAI	P
	n = 79	n = 1075	
Mean (SD) age (year)	73 (16.3)	69 (19.5)	0.12
Gender (male)	44 (55.7)	628 (58.4)	0.64
Mean (SD) BMI (kg/m^2^)	23.9 (4.4)	24.9 (5.7)	0.16
Weight loss ≥ 5%*	15 (19.0)	163 (15.2)	0.058
Ward speciality			
Psychiatry / Long term facility	41 (51.9)	710 (66.1)	
Surgery	17 (21.5)	145 (13.5)	0.03
Medicine	21 (26.6)	220 (20.5)	
Diagnosis category^†^			
Internal medicine	22 (27.8)	128 (11.9)	
ENT- Nervous system	18 (22.8)	273 (25.4)	
Obstetrical—Urogenital	12 (15.2)	99 (9.2)	<0.0001
Orthopedic	11 (13.9)	174 (16.2)	
Thorax	8 (10.1)	175 (16.3)	
Psychiatry	5 (6.3)	202 (18.8)	
HCAI			
Urinary tract infection	24 (30.4)	-	-
Lower respiratory tract infection, including pneumonia	18 (22.8)	-	-
Surgical site infection	15 (18.9)	-	-
Bloodstream infection	9 (11.4)	-	-
Other infection types^‡^	9 (11.4)	-	-
Gastrointestinal system infection	3 (3.8)	-	-
Time from admission to the day of prevalence^§^ (days), median (IQR^¶^)	26 (12–46)	19 (8–58)	0.28
Length of hospital stay (days), median (IQR^¶^)	47 (28–89)	33 (16–76)	0.004
Hospital mortality	7 (8.9)	33 (3.1)	0.016

Values are stated as numbers (percentages) unless stated otherwise.

*Missing data, n = 405.

^†^Missing data, n = 21. ‘Internal medicine’ includes endocrine, gastrointestinal, haematological, and skin diseases. ENT, ear-nose-throat. ‘Thorax’ includes heart, lung, and vascular diseases.

^‡^ ‘Other infection types’ include cardiovascular system infection, eye, ear, nose, throat or mouth infection, reproductive tract infection, and skin and soft tissue infection. HCAI categories were defined according to the Centres for Disease Prevention and Control definitions.

^§^ ‘Time from admission to the day of prevalence’ is the time period between hospital admission and the day of the survey.

^¶^ ‘IQR’ is the interquartile range of the median.

**Fig 2:**
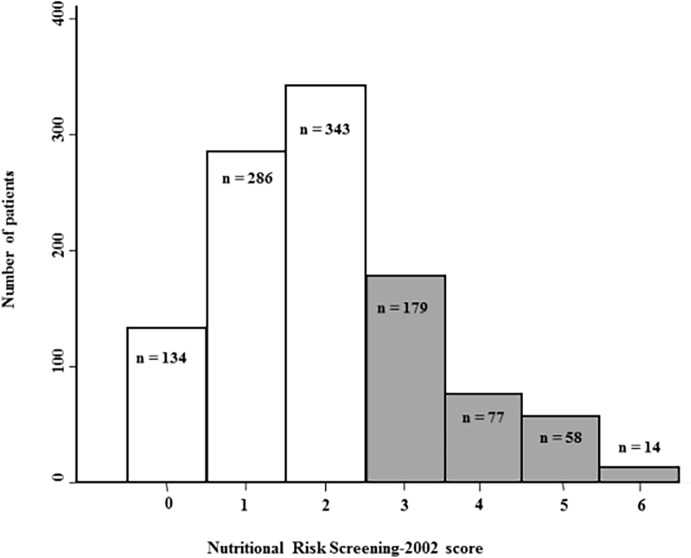
Distribution of the Nutritional Risk Screening-2002 score in the study population (n = 1091). Patients with score ≥3 are at nutritional risk (grey bars).

A total of 30.1% of patients (328/1091) were at nutritional risk according to the Nutritional Risk Screening-2002 score. Fig 2 shows the distribution of the Nutritional Risk Screening-2002 score. The proportion of patients at nutritional risk (Nutritional Risk Screening-2002 score ≥3) was not significantly different between patients with HCAI and non infected patients (26/73 (35.6%) *vs*. 302/1018 (29.7%), P = 0.28) (Table 2). The proportion of patients with Nutritional Risk Screening-2002 missing data was not significantly different between both groups (57/1075 (5.3%) *vs*. 6/79 (7.6%), P = 0.33).

**Table 2:** Nutritional Risk Screening (NRS)-2002 score and measured energy intake, according to the presence or absence of healthcare-associated infections (HCAI).

Variables	Presence of HCAI	Absence of HCAI	P
NRS-2002 score ≥3—n (%)*			
Yes	26 (35.6)	302 (29.7)	0.28
No	47 (64.4)	716 (70.3)	
Median (IQR^†^) measured energy intake—% of predicted needs^‡^	95.2 (66.1–136.6)	107.6 (82.5–136.8)	0.034
Measured energy intake ≤ 70% of predicted energy needs—n (%)^‡^			
yes	20 (30.3)	139 (14.5)	0.002
no	46 (69.7)	819 (85.5)	

Predicted energy needs are calculated as 110% of Harris-Benedict formula.

* Nutritional Risk Screening-2002 score is calculated in 1091 patients.

^†^ ‘IQR’ is the interquartile range of the median.

^‡^Energy intake is available in 1024 patients.

**Fig 3:**
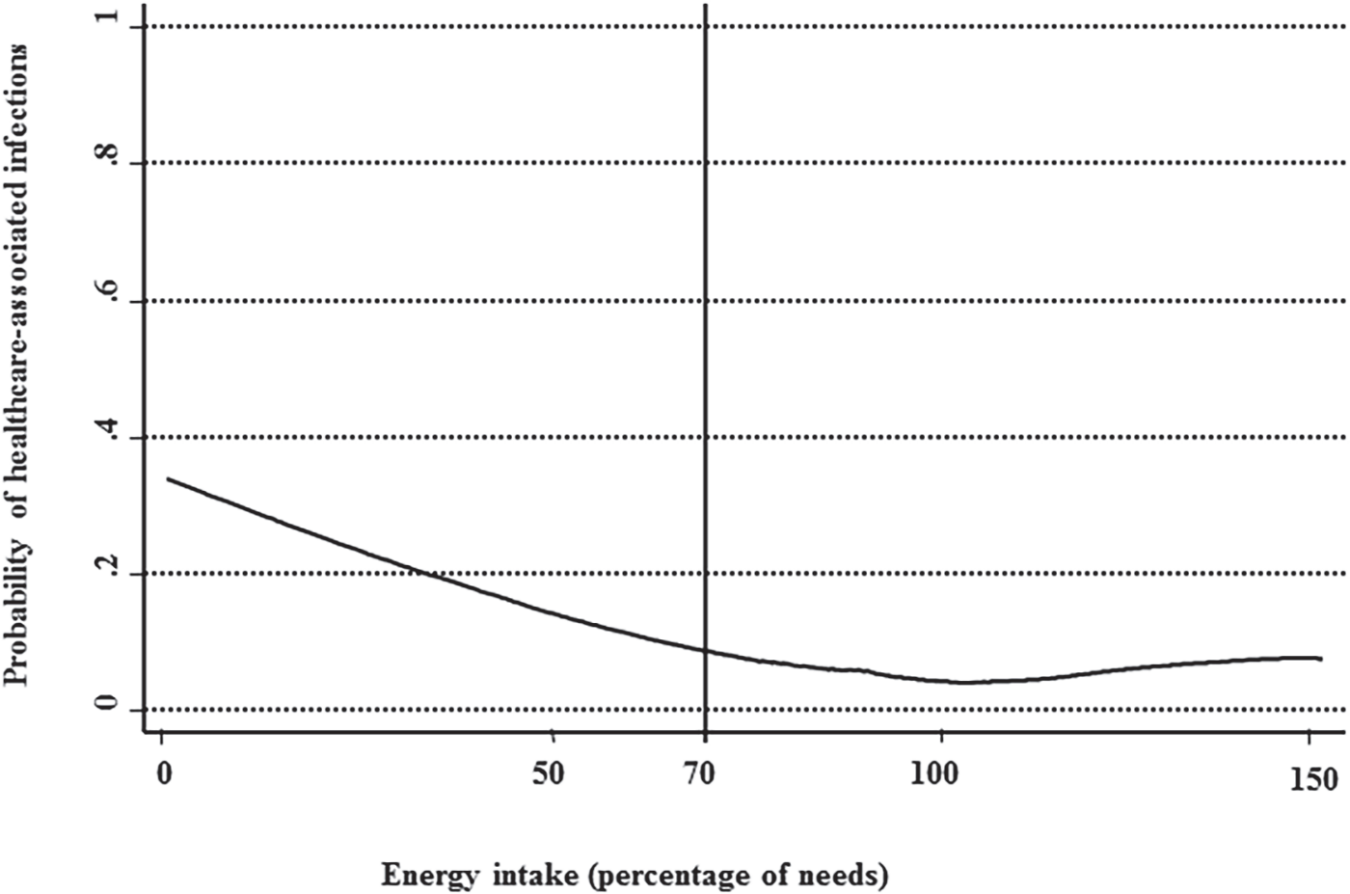
Probability of healthcare-associated infections according to the measured energy intake (expressed as % of predicted energy needs. Predicted energy needs are calculated as 110% of the Harris-Benedict formula. The Fig 3 shows that the probability of healthcare-associated infection is high when measured energy intake is ≤ 70% of predicted energy needs according to the locally weighted scatterplot smoothing graphical procedure.

The probability of HCAI was the highest when measured energy intake was ≤ 70% of predicted energy needs (Fig 3). Overall, 159/1024 (15.5%) of the patients had measured energy intake ≤70% of predicted needs. The proportion of patients with a measured energy intake ≤ 70% of predicted needs was higher in the presence of HCAI than in its absence (Table 2). The proportion of patients having measured protein intake ≤70% of predicted needs was not different in the absence (n = 343, 34%) or presence (n = 31, 43%) of HCAI (P = 0.12).

In the multivariate analysis, measured energy intake ≤ 70% of the predicted needs was associated with increased HCAI prevalence (odds ratio 2.26, 95% confidence interval, 1.24 to 4.11, P = 0.008), as well as the moderate severity of the disease (Table 3). The absence or the presence of cancer was not associated with HCAI. Using the same multivariate analysis model (data not shown), measured protein intake ≤ 70% of predicted needs was not associated with HCAI prevalence (odds ratio 0.83, 95% confidence interval 0.48 to 1.42, P = 0.49). According to our study hypothesis that increasing energy intake from ≤ 70% to >70% of predicted energy needs would have reduced the number of HCAI, a reduction of 511.6 HCAI would be expected in all the Geneva University Hospital adult patients hospitalized in acute care departments in 2012 (Table 4). It would result in a money saving of 7.2 to 7.8 million US dollars (Table 4).

**Table 3:** Multivariate logistic analysis for parameters associated with healthcare-associated infections.

	Odds ratio	[95% CI]	P
Age (<70 *vs*. ≥70 years)	0.98	[0.56–1.73]	0.96
Gender (male *vs*. female)	0.95	[0.54–1.66]	0.85
Time from admission to the day of prevalence* (day)	1.00	[0.99–1.00]	0.29
Subsections of NRS-2002 score:			
Impaired nutritional status—absent *vs*.		1	
Mild (score 1)	8.31	[0.69–100.56]	0.09
Moderate (score 2)	0.57	[0.19–1.63]	0.29
Severe (score 3)	1.33	[0.56–3.14]	0.52
Severity of disease^†^ —absent *vs*.		1	
Mild (score 1)	1.95	[0.86–4.39]	0.11
Moderate (score 2)	3.38	[1.49–7.68]	0.004
Medicine *vs*.		1	
Surgery	1.17	[0.55–2.49]	0.68
Rehabilitation-Psychiatry-Long term facility	0.83	[0.42–1.65]	0.60
Cancer (presence *vs*. absence)	0.73	[0.31–1.73]	0.47
Measured energy intake ≤70% (yes *vs*. no)	2.26	[1.24–4.11]	0.008

Predicted energy needs are calculated as 110% of Harris-Benedict formula.

CI, confidence interval.

* ‘Time from admission to the day of prevalence’ is the time period between hospital admission and the day of the survey.

^†^Score 3 for the severity of disease was integrated in the score 2 since only 4 patients had a score of 3.

**Table 4:** Estimation of money saving based on the hypothesis that increasing energy intake from ≤ 70% to >70% would have reduced the number of healthcare-associated infections (HCAI).

	Study population N = 1024	Simulated 2012 Geneva University Hospital acute care adult patients N = 45159
Proportion of HCAI in patients ≤ 70% energy needs (A)	20/159 (12.6%)	882/7013 (12.6%)
Proportion of HCAI in patients > 70% energy needs (B)	46/865 (5.3%)	2029/38146 (5.3%)
Expected reduced rate of HCAI [(B–A)/A]	–58%	–58%
Expected number of HCAI*	8.4	370.4
Expected number of saved HCAI	11.6	511.6
Expected money saving (million US dollar)^†^	0.16–0.18	7.2–7.8

The financial simulation was transposed from our study population to the whole Geneva University Hospital adult patients hospitalized in acute care departments in 2012 (source: 2012 Swiss-DRG data from the Department of Informatics of the Geneva University Hospital).

* Number of HCAI expected by reducing by 58% the proportion of HCAI if a nutritional intervention would have covered > 70% instead of ≤ 70% of their energy needs in the study population and in the 2012 Geneva University Hospital acute care adult patients.

^†^ The estimation of money saving was calculated according to CDC data [24] based on the low and high estimates of average HCAI attributable costs, 14,000 and 15,300 US $/patient, respectively.

## Discussion

Our study shows that measured energy intake ≤ 70% of predicted energy needs is related to increased HCAI prevalence. To our knowledge, this is the first study to report an association between HCAI and measured insufficient nutritional intake in a general hospital population.

At hospital admission, patients at nutritional risk are insufficiently identified, mainly due to the fact that validated scores of nutritional screening (e.g. Nutritional Risk Screening-2002, Mini Nutritional Assessment) are time consuming and lack of simplicity. These scores require the collection of several parameters, including body mass index and weight loss. Interestingly, in our model, body mass index and weight loss were not related to HCAI. Therefore, this study indicates that the assessment of dietary intake could be a mean to identify patients at risk of HCAI, and that dietary intake should be systematically assessed in patients with HCAI. These findings are in line with previous studies showing a relation between insufficient energy intake and poor clinical outcome in hospitals [8,9,12]. The European multicentre observational survey NutritionDay has demonstrated that eating a quarter of provided meals only was an independent risk factor for hospital mortality [8]. In 3122 Australian hospitalised patients, insufficient dietary intake was associated with longer hospital stay, more readmissions, and higher mortality [9]. In the study by Tangvic et al, decreased dietary intake was associated with a doubling of one year mortality [10]. Previous studies showed that a timely nutritional intervention could reduce HCAI. Improving energy intake with supplemental parenteral nutrition to cover 100% of energy needs in critically ill patients after an initial phase of enteral nutrition was effective in reducing by 29% HCAI incidence [13]. The incidence of postoperative infectious complications was shown to be significantly reduced by the enteral administration of immunemodulatory nutrients in oncologic patients undergoing abdominal surgery [14].

Our cut-off of 70%, below which the risk of HCAI is increased, was identified by locally weighted scatterplot smoothing (lowess) graphical procedure (Fig 3). This threshold is in compliance with actual recommendations of academic societies to detect nutritional risk or initiate a nutritional support. The French Society of Clinical Nutrition and Metabolism (SFNEP) recommends the use of an analogue visual scale evaluating patient’s alimentation to detect nutritional risk in oncologic patients [25]. Scores of analogue scale were shown to be tightly correlated with dietary intake assessed by a 3-day dietary record [26]. A score below 7/10 was correlated with the risk of malnutrition in hospitalised patients and out-patients [26]. In surgical patients, the European Society for Clinical Nutrition and Metabolism (ESPEN) and SFNEP recommend to initiate a postoperative nutritional support if patients do not meet 60% of their predicted energy needs during the ten days following surgery [27,28]. On the contrary, in the United Kingdom, the National Institute for Health and Care Excellence (NICE) recommends initiating a nutritional support only for patients eating nothing or almost nothing per mouth[29]. The results presented here suggest that the intervention should concern patients with energy intake ≤ 70% of predicted needs. Our prevalence of patients with nutritional risk (30.1%) is similar with a recent study performed in Norwegian hospitals (29%) [10].

In Switzerland, the prevalence of HCAI is between 9.8% and 13.5% by the period prevalence methodology [30]. In the present study, the prevalence of HCAI was 6.8%. This lower prevalence may be due to the fact that only patients eating three meals per day were included. They are supposed to have less severe diseases. Indeed, patients receiving exclusive enteral and parenteral nutrition, e.g. intensive care unit and surgical patients, are those with the higher risk of HCAI, whom were excluded from the study. In our study, a relation between a moderate severity of the disease and an increased HCAI prevalence was found, as previously reported [30].

### Strengths and weaknesses

This study is part of a general audit about the nutritional status performed at the Geneva University Hospital every four years since 1999 [17,18]. Consequently the methodology has been tested and validated. The measurement of nutritional intake by recording real dietary intake of all meals strengthens the data of energy intake, which are usually based on the subjective evaluation of the patients’ past week dietary intake. The diagnosis of HCAI was made using a standardized methodology, which was published and is widely used [21]. Our study has several limitations. First, the study used a one-day cross-sectional methodology, and did not prospectively assess dietary intake during the entire hospital stay. However, in a large hospital such as the Geneva University Hospital, a one-day prevalence survey offers a manageable and reliable method to assess the overall nutritional situation. Prospective studies are needed to assess whether changes in energy intake during the hospital stay (worsening vs. improvement) could have an impact on clinical outcome. We cannot exclude that HCAI impact on dietary intake and that anorexia could be a consequence of the HCAI. Regarding the important possible outcomes of insufficient dietary intake, assessing and improving dietary intake would avoid or limit HCAI associated with insufficient dietary intake. Second, non-infectious complications were not recorded. This does not interfere with the findings that energy intake below 70% can promote HCAI but other risk factors of interest could have been identified. Third, the Nutritional Risk Screening-2002 score could not be performed in all analysed patients (missing data for 63 patients). If our excluded patients had been considered, insufficient energy intake would have been higher. Therefore, our data underestimated the real proportion of patients with insufficient energy intake. Finally, this study highlights the need to implicate both infectiologists and nutritionists in optimizing clinical outcomes and healthcare-costs, specifically related to HCAI. Nonetheless the study was not interventional, and it cannot be concluded that a dedicated intervention to increase energy intake could reduce the prevalence of HCAI.

### Clinical implications

Insufficient dietary intake is one of the main risk factors for malnutrition, which in turn is related to HCAI, increased length of stay and higher costs [1,6,9,10,31]. HCAI have been identified as major challenge in hospitals, and many studies have reported successful prevention strategies addressing best practice in patient care [32–37]. Identifying patients with malnutrition and improving dietary intake is a novel strategy in HCAI prevention. Given the important proportion of patients with insufficient dietary intake in our study (15.5%), many patients would benefit from such a prevention programme. Future intervention studies must confirm our findings and estimate the level of improvement to be obtained by improving dietary intake in hospitalised patients. The early detection of insufficient dietary intake in patients would also have a financial impact as it will increase the Diagnosis Related Groups invoicing of hospital stays through the boosted identification of malnutrition. Detecting and preventing insufficient dietary intake could have massive financial impact. First, as insufficient dietary intake is the main cause of malnutrition, early detection of insufficient dietary intake would improve the rate of malnutrition diagnoses. In our hospital, in 2012, malnutrition was coded as a Diagnostic Related Group (DRG) for 553 hospitalizations out of a total of 45,159 DRG-coded hospitalizations (source: 2012 Swiss-DRG data from the Department of Informatics of the Geneva University Hospital). After investigations, malnutrition should have been coded for 5,265 hospitalizations meaning that 4712 hospitalizations have not been coded, which would have generated an extra income higher than 3.3 million dollars. In a Croatian study, Benković et al estimated that the costs of malnutrition in patients with disease-related malnutrition were over 97 million euros per year (i.e. 3.38% national health cost) [6]. Second, improving dietary intake could have financial impact by reducing the rate of HCAI. The financial simulation in all the Geneva University Hospital adult patients hospitalized in acute care departments in 2012 suggest that a reduction of 511.6 HCAI in malnourished patients could allow a money saving between 7.2 and 7.8 million US dollars (Table 4) [24]. A prospective interventional study is needed to verify this hypothesis and this financial projection. Therefore, the early detection of insufficient dietary intake would have significant clinical and economic impacts by reducing HCAI rate and hospital healthcare-related costs, respectively. In addition, the exhaustive billing of malnutrition would increase the hospital financial resources.

## Conclusion

This cross-sectional study conducted in a large population of hospitalised patients shows that insufficient measured energy intake ≤ 70% of the predicted needs and the moderate severity of the disease were associated with HCAI. This finding could suggest that insufficient dietary intake could be a risk factor of HCAI, without excluding reverse causality. Future randomized trials should aim at demonstrating that an early nutritional intervention in hospitalised patients identified with insufficient dietary intake would decrease the incidence of HCAI, and that this strategy is cost-effective. Nutritional Risk Screening-2002 score is a validated nutritional screening tool, but its validity to identify patients at risk of HCAI remains to be determined in prospective studies.
